# An Overview of the Genetic Mechanisms of Colistin-Resistance in Bacterial Pathogens: An Indian Perspective

**DOI:** 10.7759/cureus.78800

**Published:** 2025-02-09

**Authors:** Kajal S Yadav, Kailas Datkhile, Satyajeet Pawar, Satish Patil

**Affiliations:** 1 Department of Microbiology, Krishna Institute of Medical Sciences, Krishna Vishwa Vidyapeeth (Deemed to be University), Karad, IND; 2 Department of Allied Sciences, Krishna Institute of Medical Sciences, Krishna Vishwa Vidyapeeth (Deemed to be University), Karad, IND

**Keywords:** colistin resistance, klebsiella pneumoniae, mcr-1, mcr genes, resistance mechanisms, two component system

## Abstract

Colistin resistance in bacteria is a growing global issue, given its role as a critical last-resort antibiotic, particularly for treating Gram-negative bacterial infections. Pathogens adopt multiple resistance mechanisms, mediated either by plasmids or chromosomal changes. Some of the most frequently observed strategies include the occurrence of plasmid-borne mobile colistin resistance (mcr) genes, enhanced efflux pump activity, mutations in the regulatory systems, and alterations in the lipid A structure. This article provides an overview of the studies investigating the genetic mechanisms underlying colistin resistance in nosocomial Gram-negative bacteria from India. A total of 37 studies were identified through online searches across various databases, including PubMed, ScienceDirect, and Web of Science. These studies were reviewed to examine bacterial species and their mechanisms of colistin resistance. Over 26 (70.27%) studies were focused on *Klebsiella pneumoniae*. The most commonly reported mechanism of colistin resistance involved mutations in the two-component systems pmrAB and phoPQ. Plasmid-mediated colistin-resistant mcr genes were identified in 22 studies (18.18%). Four studies reported the overexpression of efflux pump genes as a mechanism of colistin resistance. This article provides a comprehensive summary of these studies, emphasizing the presence of diverse resistance mechanisms across various pathogens. It underscores the necessity for future genomic research on a broader range of pathogens to investigate the prevalence of different mechanisms of colistin resistance in the various regions of India.

## Introduction and background

The misuse of antibiotics can drive adaptive evolution in bacteria, making antibiotic resistance in pathogens a critical global concern that places immense strain on public health systems [1,2]. Pathogenic bacteria develop or adapt various mechanisms of antibiotic resistance that enable them to survive antibiotics. Notably, they can either possess innate resistance or acquire it from the neighboring bacteria [3]. Pathogens that develop drug resistance have a competitive advantage over antibiotic-sensitive bacteria [4]. Drug-resistant bacteria can collaborate with co-existing bacteria and acquire resistance to additional antibiotics, thereby becoming multidrug-resistant [5]. They may employ various mechanisms of resistance against a specific drug [6,7]. Thus, it is important to investigate the exact mechanisms of drug resistance in pathogenic bacteria and their prevalence.

Colistin is considered a 'last-resort' antibiotic and is utilized in severe pathophysiological conditions caused by Gram-negative bacteria that are multidrug-resistant or extensively drug-resistant [8]. It has a narrow antibacterial spectrum, primarily targeting common Gram-negative bacteria within the Enterobacteriaceae family, including the Klebsiella species, *E. coli*, Citrobacter species, Shigella species, Enterobacter species, and Salmonella species [9]. Colistin exerts its antibacterial activity on the outer cell membrane of Gram-negative bacteria, which is characterized by a lipopolysaccharide (LPS) layer that restricts the entry of hydrophobic molecules and antibiotics [10]. As the polycationic peptides colistin or polymyxins interact with lipid A, a hydrophobic component of the LPS layer, its structure is disrupted through the electrostatic displacement of divalent cations. This disruption permeabilizes the outer LPS membrane, leading to the leakage of the intracellular contents and ultimately causing bacterial death [11]. Additionally, colistin functions as an anti-endotoxin. The lipid A component of LPS acts as an endotoxin in Gram-negative bacteria. Colistin inhibits this activity by binding to and neutralizing LPS molecules [11].

Since its discovery, colistin has been used in farm animals across Asian countries such as India, China, Pakistan, and Bangladesh [12,13]. The rise of colistin resistance in these regions has become a major global concern [9], as resistance can spread horizontally via conjugative plasmids or vertically through chromosomal mutations [14]. Some bacteria are naturally resistant to colistin, while others acquire it [15]. The primary strategy bacteria use for colistin resistance involves alterations in the LPS, which are mediated by genes responsible for maintaining electrostatic balance, capsule formation, and the overexpression of efflux pump genes. Additionally, several genes and operons, such as pmrC, play a role in colistin resistance through LPS modification. Mutations in the two-component regulatory systems (TCS), including pmrAB and phoPQ, are also implicated in colistin resistance [11,16]. A recent emerging mechanism of colistin resistance involves a decrease in the binding affinity of colistin to the outer membrane, mediated by plasmid-borne mobile colistin-resistance (mcr) genes, which are responsible for adding phosphoethanolamine to lipid A [17]. Together, these mechanisms enable bacteria to resist colistin and survive treatment. Recent reports indicate that colistin resistance is rising globally, with new mechanisms of resistance continuing to be discovered [15]. Moreover, novel mutations in colistin-resistant bacteria and variants of colistin resistant genes are being reported [18-21]. Analysis of the global prevalence of colistin-resistant genes in bacteria shows that their frequencies vary by geographic region and bacterial isolate [11,22,23]. Additionally, recent reviews have focused on colistin resistance in specific organisms or mechanisms of resistance [22-25]. Given the need for timely updates on emerging colistin resistance in pathogenic bacteria, we examined its trend in India.

## Review

Article search strategy

In this article, we reviewed studies on colistin resistance in nosocomial pathogenic bacteria and their genetic mechanisms from India. To gather relevant information, we searched for research articles and short papers on colistin resistance in English through databases such as PubMed, ScienceDirect, Embase, and Web of Science. We also explored individual publisher databases, including Springer, BioOne, Frontiers, Nature, and Medknow. Additionally, we examined the references and citations of recently published articles. To find the relevant articles, we used keywords such as "colistin resistance," "bacteria" or "microbiology," and "India," employing "and" and "or" Boolean operators. After an extensive search, we identified a total of 37 studies on the genetic mechanisms of colistin resistance in nosocomial pathogens. We reviewed the colistin-resistant bacterial pathogens mentioned in these studies and highlighted the associated genetic or molecular mechanisms.

Colistin resistance in Indian pathogens

**Figure 1:**
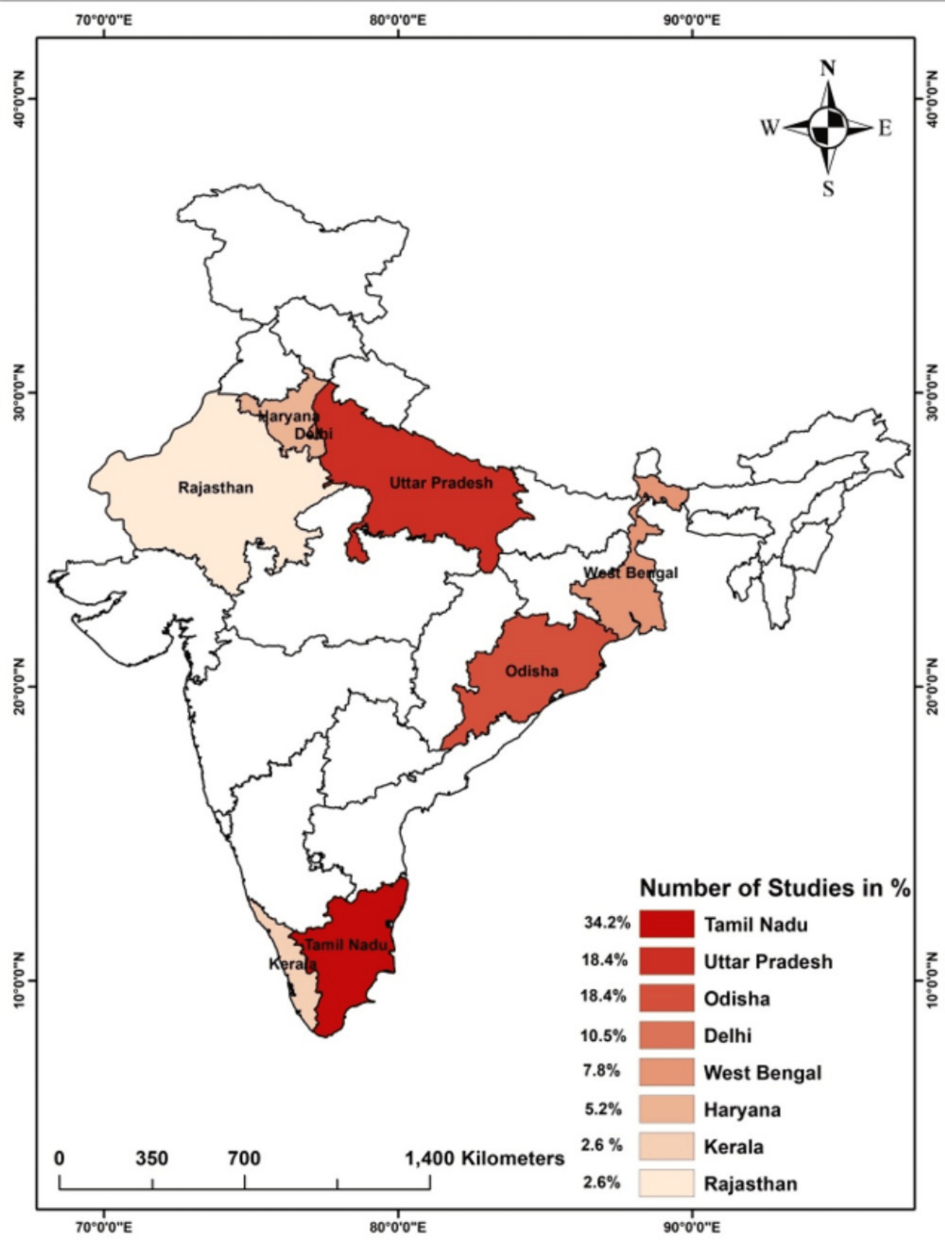
Map showing the percentage of the Indian studies reported from different states mcr: Mobile colistin-resistant gene

Research on colistin resistance in pathogenic bacteria began in India shortly after the discovery of plasmid-mediated mcr genes in China [26]. The first study investigating the molecular mechanism of colistin resistance was conducted on *K. pneumoniae* and it did not find mcr genes in any of the isolates [19]. However, the study did report mutations in the mgrB gene and other genes associated with lipid A modification [19]. Among the studies conducted in India, 13 (35%) were from the southern state of Tamil Nadu, followed by the northern state of Uttar Pradesh (Figure 1).

**Figure 2:**
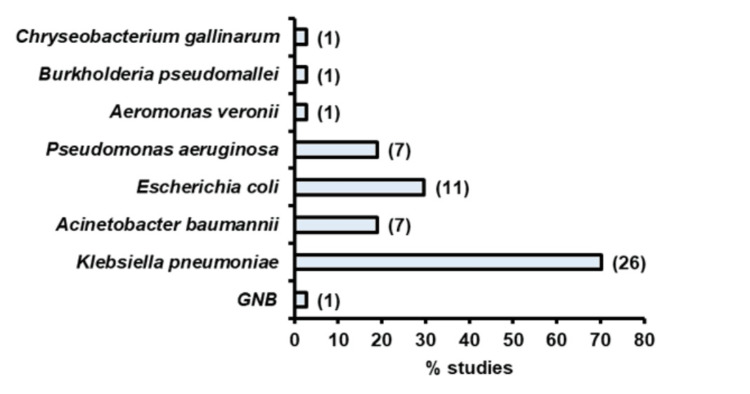
Proportion of the studies that reported (total 37) colistin resistance in different pathogens GNB: Gram-negative bacilli

Seventeen of these studies used genomic tools to investigate the genetic mechanisms of colistin resistance, while 20 studies employed PCR-based techniques to detect the presence of antibiotic resistance genes or specific genes. The majority of studies focused on *K. pneumoniae*, followed by *E. coli*, *P. aeruginosa*, and *A. baumannii *(Figure 2). One study from Odisha did not specify the bacterial species involved [27].

Occurrence of mcr genes

A total of 29 studies explored the occurrence of mcr genes in colistin-resistant bacteria, with 27 of these focusing on *K. pneumoniae* isolates. Among them, only nine studies detected various mcr genes in bacteria, and just five studies identified mcr genes in *K. pneumoniae.* Notably, Singh et al. (2021) [28], and Singh et al. (2018) [29] reported the presence of the mcr-1 gene in 86.4% and 19.4% of *K. pneumoniae* isolates, respectively. Further studies on *K. pneumoniae isolates* by Bir et al. (2022) [30], Irusan et al. (2021) [31], and Roy et al. (2020) [32] reported the mcr-1 gene in 6.7% of isolates [30], mcr-5 gene in 1.58%, and mcr-1 gene in a single isolate, respectively. In the case of *E. coli* isolates, Palani et al. (2020) [33] reported the presence of mcr-1 in 66.67% of them and Roy et al. (2020) [32] reported the mcr-1 gene in one. Singh et al. (2018) and Pathak et al. (2020) reported mcr-1 in 75% and 80% of *P. aeruginosa* isolates, respectively [29,34]. Rahman et al. (2020) identified mcr-1 in 20% of *A. baumannii *isolates [35]. Azam et al. (2021) [36] did not find the mcr-1 to mcr-8 genes in *K. pneumoniae* isolates, while Sharma et al. (2021) [37] also reported the absence of mcr-1 to mcr-5 genes in their* K. pneumoniae* isolates. Additionally, Mitra et al. (2020) [27] reported the mcr-1 gene in 6.25% and mcr-2 in 25% of the Gram-negative isolates.

Other genes involved in colistin resistance

In the present analysis, we encountered a total of 22 studies exploring mechanisms of colistin resistance other than plasmid-mediated resistance (mcr) in four bacterial species: *K. pneumoniae*, *Chryseobacterium gallinarum*, *P. aeruginosa,* and *A. baumannii*. Among these studies, 20 were performed on *K. pneumoniae,* and one study each was focused on the remaining species [21,38,39]. All the studies, except Naha et al. 2020 [40], explored the involvement of TCS in colistin resistance. Colistin resistance due to lipid modifications was reported in nine studies while mutations in efflux pumps were studied by four authors. The mutations in pmrABC and mgrB systems were reported in a total of 15 studies followed by phoPQ system. Interestingly, genomic studies exploring the involvement of TCS were more in number as compared to the studies targeting a specific gene using the PCR technique (Table 1).

**Table 1:** Mechanisms of colistin resistance in Gram-negative bacteria based on genetic alterations and efflux pumps TCS: Two-component regulatory systems

Organism (Source study)	TCS	Lipid A	Efflux pump
K. pneumoniae	-	-	-
Singh et al., 2021 [28]	mgrB	-	AcrAB, mdtK
Nirwan et al., 2021 [ 41]	pmrAB, phoPQ, mgrB	-	-
Azam et al., 2021 [36]	phoP, pmrA, pmrB, mgrB, pmrC	-	-
Mathur et al., 2018 [42]	mgrB, pmrB, phoP and phoQ	eptA, arnT	-
Mathur et al., 2019 [43]	phoPQ, pmrB, mgrB	eptA, arnTm	-
Palani et al., 2020 [33]	mgrB	-	-
Shankar et al., 2019 [44]	mgrB, phoP, phoQ	-	-
Pragasam et al., 2017 [19]	mgrB, phoPQ, pmrABD	-	-
Sharma et al., 2021 [37]	pmrAB	lpxAD	-
Bir et al., 2022 [30]	pmrAB, pmrC, phoPQ	pmrC, pmrE, arnBCT, arnA_FT, pagP	-
Naha et al., 2020 [40]	-	-	AcrAB-TolC
Naha et al., 2022 [18]	pmrABC, phoQ, mgrB, crrAB	lpxBM, pagP	AcrAB-TolC, ramR, macB
Talat et al., 2024 [39]	mgrB, pmrB, eptA/pmrC, eptB	arnT, ampA	-
Kumar et al., 2016 [45]	mrgR, mrgS, mrgB	-	-
Kumar et al., 2018 [46]	mgrB	-	-
Kaza et al., 2024 [47]	mgrB	-	-
Palani et al., 2020 [33]	mgrB	-	-
Basu et al., 2024 [48]	pmrB	-	-
Sahoo et al., 2023 [49]	pmrB, eptB	-	-
Khurana et al., 2023 [50]	pmrB, mgrB	-	KpnEFGH, RND efflux pump
Chryseobacterium gallinarum	-	-	-
Gaur et al., 2022 [38]	pmrB	lpxD	-
P. aeruginosa	-	-	-
Talat et al., 2024 [ 39]	pmrB, cprR, cprS, parS	eptA, arnA, basS, basR	-
A. baumannii	-	-	-
Vijayakumar et al., 2024 [21]	pmrABC, eptA/pmrC	lpxD	-

Previous reports suggest that *K. pneumoniae* is the most prevalent pathogen responsible for colistin resistance [24,51]. The observations of the current study are in accordance with this [24,51]. However, several studies reported that other Gram-negative bacteria also developed or evolved with colistin resistance [52]. Further studies need to be undertaken to explore the mechanisms in other pathogenic bacteria.

These findings suggest that the pathogens in India exhibit diverse mechanism of colistin resistance. The most common mechanisms of colistin-resistance are the mutations in the TCS including pmrAB, mgrB, and phoQ. Previous studies [25] observed that mutations in mgrB gene are the common in *K. pneumoniae* isolates followed by pmrB, phoQ, and phoP. These observations suggest that the TCS are the major obstacles in the treatment of colistin-resistant pathogens. They are sensitive to external stimuli, alter gene expression accordingly, and help bacteria adapt. These TCS are composed of histidine kinase and response regulator proteins. Their involvement is alarming since growing mutations in their genes are responsible for the colistin resistance. Further, components of the TCS in *K. pneumoniae* can be targeted for the future antibacterial therapy [53,54].

The use of modern genomic techniques is important for investigating the different mechanisms of colistin resistance. Genomic techniques also help in detecting the variants in the colistin resistant genes and different mutations in a particular gene [18,20,41]. They are particularly crucial in the case of emerging resistance to last-resort antibiotics.

Plasmid-mediated mcr genes resistance is an emerging concern due to their possible for horizontal gene transfer [55]. Currently, mcr1 to mcr10 genes play a role in colistin resistance by adding phosphoethanolamine (PEtN) to lipid A [56]. Mcr genes have some conserved amino acid sequences, but they have originated from diverse backgrounds [56]. They have been found in several Gram-negative bacteria within the Enterobacteriaceae family [57]. However, the present overview infers that mcr genes are rarely found in nosocomial pathogens in India and highest number of these genes were reported in *K. pneumoniae*. These inferences are in agreement with the previous reports that the global prevalence of mcr genes is 4.7% [16].

The most striking observation in the current study is the limited studies on the genetic mechanisms of colistin resistance in India. India is an extremely diverse country as far as biogeography and ethnicity is concerned. These parameters can also influence the prevalence of resistance in pathogenic bacteria [58,59]. The mechanisms of colistin resistance have been reported from eight states of India, focusing majorly on a single pathogen. These observations demand multiple studies on different pathogens from diverse biogeographical zones. 

## Conclusions

In this article, we have confirmed that colistin resistance in various pathogenic bacteria is governed by multiple genetic mechanisms. Among these, the most prevalent mechanism observed in Indian bacterial pathogens involves mutations in the TCS, which play a crucial role in modulating resistance. Genomic studies have been instrumental in providing a comprehensive understanding of the genetic and molecular mechanisms underlying colistin resistance. These studies enable the identification of key mutations, regulatory pathways, and resistance determinants that contribute to reduced susceptibility to colistin. Such insights are essential for developing targeted interventions to combat antimicrobial resistance.

The findings of this study underscore the urgent need for the development of effective strategies to address the rising threat of colistin resistance. Given the critical role of TCS in mediating resistance, future efforts should focus on exploring novel therapeutic approaches, enhancing surveillance programs, and implementing stringent antimicrobial stewardship policies. A deeper understanding of these genetic mechanisms will aid in the formulation of precision-based strategies to mitigate the spread of colistin-resistant bacterial strains and preserve the efficacy of this last-resort antibiotic.
